# Nurses’ Experiences as Care Providers for Refugees in Emergency and Critical Care in Jordan: A Qualitative Interview Study

**DOI:** 10.1177/23333936211056932

**Published:** 2021-11-10

**Authors:** Maja Backlund, Sepideh Olausson

**Affiliations:** 1 Sahlgrenska Academy, 70712 Institution for Nursing Science and Health at Gothenburg University, Gothenburg, Sweden

**Keywords:** nursing, qualitative research, postcolonial, healthcare equity, refugee crisis, health disparities, Jordan

## Abstract

During the global refugee crisis of the 2010s, hundreds of thousands of Syrians fled to Jordan. As displaced Palestinians have had refugee status for several decades in Jordan already, this study aimed to explore nurses’ perceptions of caring for Palestinian and Syrian refugees within the context of critical and emergency care. The qualitative design was executed through twelve semi-structured interviews with nurses working in refugee camps and public hospitals. Three main themes were identified describing the nurses’ empathetic understanding of the refugees’ situation, various challenging factors, as well as different aspects of the opportunities that they perceived in critical care and emergency care. The experiences of publicly employed nurses generally differed from those working in the camps. In addition, the findings indicate the importance of further research conducted locally, as it suggests several elements that have a negative impact on the quality of advanced healthcare for refugees.

## Introduction

Globally, more people were driven from their homes by armed conflicts during the 2010s than at any time since the Second World War (Doocy et al., 2015). What is called “the refugee crisis” not only is a personal disaster for those who flee, it has had a significant impact on the countries neighboring conflict areas. There is a significant variation to the extent different countries are affected by the conflicts. Developing countries receive the most refugees (European Commission, n.d.; Doctors Without Borders, 2021) but are the least equipped to handle such influx. Jordan is among these countries, being poor in resourcesand rich in refugees. In this study, a postcolonial approach was adopted to actively consider the history of racialization, subsequent colonial relations, and the effects of that discourse on the conditions of healthcare in the area (Kirkham & Anderson, 2002; Mohammed, 2006).

Nurses have a responsibility to strive to meet the health and social needs of the public, especially the most vulnerable as emphasized by International Council of Nurses (ICN, 2012). Nurses should actively seek knowledge about patients’ living conditions or life world. Furthermore, social justice is an explicit mission for the nursing profession, through the responsibility of ensuring equity in healthcare access (ICN, 2015). Nurses’ perceptions of refugee patients’ living conditions therefore could be considered an important subject to investigate. As nurses, we are expected to include specific aspects of a refugee patient’s situation in their assessments and action plan (Squires, 2016). For example, mental trauma from the events leading to life in refuge often presents once refugees have started to settle in their host countries, and thus handling this process requires appropriate training and experience.

### Jordanian Refugee Policy

Jordan is one of the countries that have not ratified the UN refugee convention of 1951, but the country has historically supplied refugees with free healthcare and education (Shiblak, 1996). Other conventions ratified by Jordan do protect refugees to some extent (Dadzie, 2017). Notably, children are not among the groups protected by any conventions that Jordan adheres to. Dadzie (2017) also points out that Jordanian law and policies do restrict refugees’ social status. For example, the constitution limits the right to work for refugees who are considered “guests” and not citizens, since Jordan is not otherwise obligated.

The Jordanian Ministry of Health (MOH) implemented hospital fees for refugees in 2014, referring to untenable expenses (Al-Rousan et al., 2018). Refugees from then on has had to pay an amount equivalent to that required from Jordanians who are without health insurance when visiting public hospitals (Francis, 2015). This was an important development that likely impacted health equity for refugees in Jordan in a negative way (Doocy et al., 2016). Before then, healthcare was offered free of charge for all registered refugees (Lupieri, 2020). In 2018, policy change led the cost to be 2–4 times higher, about the same as the so-called foreigner rate, and then back to the “uninsured rate” in 2019. Hidden or unregistered refugees, of course, always encounter severe barriers to healthcare access when healthcare policy demands registration (Hacker et al., 2015; Martinez et al., 2015). Notably, Syrians of Palestinian descent have been denied entry to Jordan since early in the Syrian civil war and are therefore unregistered if they crossed the border (Francis, 2015).

To request tertiary care such emergency care or critical care, refugees need approval from the Exceptional Care Committee of the United Nations High Commissioner for Refugees (UNHCR) to access free services (Dator et al., 2018). The criteria are strict, and not all who perceive the need for emergency care will be approved. Moreover, it complicates the procedure which could mean that overworked staff might not have the time or knowledge to explain the referral system to refugees. Refugees trying to access public hospitals (run by MOH) on their own will be turned away.

### Palestinian and Syrian Refugees in Jordan

Over the years, Jordan has received hundreds of thousands of Palestinians seeking refuge from war and occupation (United Nations Relief and Works Agency, UNRWA, 2013). They and their descendants now number over two million people (UNRWA, 2015) within Jordan’s population of 10 million (Jordanian Department of Statistics, 2017). In addition to the former Palestinian refugee influx, the country has received many more refugees in recent years. Syrians fleeing civil war since its outbreak in 2011, and by 2014, became the second largest group of refugees in Jordan, numbering about 620,000 registered refugees (UNHCR, 2014). Both Za’atari and Azraq refugee camp were established and filling up fast by then. Due to less welcoming Jordanian refugee policy including border control, the number has remained relatively constant since; in 2021, the number is estimated to be 660,000 Syrian refugees, while other nationality refugees (Palestinians excluded) amounted to 90,550 by the end of 2020 (UNCHR, n. d.). Half of the registered Syrian refugees are female, and half are less than 17 years old (Dhaini et al., 2020). In total, approximately 1.3–1.5 million people of Syrian heritage are estimated to be living in Jordan, as many immigrated before the recent war as well (Lupieri, 2020).

About 80% of Syrian refugees live outside the camps, sharing scarce urban resources with the rest of the population. Furthermore, 80% of Syrian refugees were already living under the national poverty line before the COVID-19 Pandemic (Human Rights Watch, 2016; UNHCR, n. d.). According to the UNHCR (n.d.) approximately 130,000 Syrian refugees lived in camps by March 2021. Camp life may become their long-term living situation, as it has for more than 18% (370,000) of the total Palestinian refugee population, who live in 10 official UNRWA camps in Jordan (Alduraidi, 2016). In the Palestinian camps, poverty was estimated at around 30% (compared to 13% for non-camp refugees and non-refugees in Jordan) back in 2008. Life in the Palestinian camps of Jordan has reported issues of poor and crowded housing, inadequate electricity, and infrastructure including sanities, safety issues, and socio-economic deprivation, all leading to a worse physical and mental health for camp residents when compared to the rest of the population (Tiltnes & Zhang, 2014). Poverty in itself is associated with lack of education, chronic health conditions (Tiltnes & Zhang, 2014), and other health problems (Mousa et al., 2010; Amara & Aljunid, 2014). Also notable is that 85% of Palestinian refugees residing in the camps hold Jordanian citizenship, compared to 96% of the Palestinian Jordanians living outside the camps in Jordan, meaning they lack legal job opportunities to a larger extent as well.

### Healthcare and Refugees in Jordan

High blood pressure, diabetes, and obesity are known health issues in the Palestinian refugee group (Amara & Aljunid, 2014). Compared to the Jordanian population in general, there is a particularly large difference regarding obesity; more than 30% percent of Palestinian refugee males and more than 50% of Palestinian refugee women in Jordan are obese compared to 10% and 16%, respectively, in the rest of the Jordanian population. Smoking is also a known health hazard for Palestinian men in particular; due to cultural norms, there is an assumed underrepresentation among women who report as smokers. Palestinian refugees in the Baqa’a and Wihdat camps have shown tendencies toward incorrect use of medications for chronic disease, overuse of antibiotics, and a tendency to seek advice from pharmacists rather than physicians (Qato, 2013). Palestinians in Jordan have a generally low self-reported health-related quality of life compared to the expected levels for other Jordanians and often present with symptoms of depression (Alduraidi, 2016).

For Syrian refugees, medication costs for chronic disease, which is prevalent in 30–50% of the population, has been reported as a significant barrier (UNRWA, 2015; Doocy et al., 2016; Dator et al., 2018). Adult Syrian refugees present with the non-communicable diseases of the typical lower-middle–income nation that Syria was before the recent war, but also communicable diseases such as tuberculosis (Saleh et al., 2018). Women’s health is an issue, especially regarding access to antenatal care, teen pregnancy, increasing maternal mortality, other obstetric complications, and sexual violence. Among children, communicable diseases are common, and the war has led to a decline in immunization rates. Psychiatric disorders (at 30%) and war related physical injuries (at 15%) are also present in the Syrian refugee group (UNRWA, 2015).

Syrian refugees in Jordan primarily seek healthcare for prevalent acute and communicable diseases, chronic diseases, and dental problems (Dator et al., 2018). They mainly seek out government facilities; 40% reported using public health centers, and 34% reported going to public hospitals according to the review. About 25% had needed emergency care and access was pointed out as an issue here; preventive and primary healthcare were reported to be more accessible than advanced services and in-patient care. Only 50% of the families interviewed thought emergency services would be available to them, but the number rises to about 80% among those who had actually had a need for it. This is a trend observed in all categories of advanced healthcare, suggesting a lack of information regarding access to these services. Mental healthcare was the least reported need at just below 10% (Dator et al., 2018), leading to the conjecture that although many have psychological issues, they are not treated for them but instead bring untreated mental health problems with them into somatic healthcare.

Transportation cost is a highly reported financial barrier to healthcare for refugees, alongside medicine expenses (Al-Rousan et al., 2018; Dator et al., 2018). Structural barriers reported according to the review by Dator et al. (2018) include long waiting times, late appointment dates, long procedures to get use of services, and long distances to health facilities. The interview study by Al-Rousan et al. (2018) points out poor livelihood, poor housing conditions and water quality, changing policies, and limited health literacy as sources of illness that the refugees (camp and no camp) themselves identified. The most notable cognitive barrier reported by both studies was discrimination by health personnel. Many of the refugees even preferred to take medicine without consulting a doctor as they did not trust the services provided to them. Al-Rousan et al. (2018) found a discrepancy here; conversely to the refugees reports of discrimination, the Jordanian healthcare personnel emphasized how much hospitality the refugee patients were received with. They did, however, express feeling overworked or even burnt out, reporting that they struggled with the provision of care due to understaffing and limited resources.

The living conditions of refugees make them particularly vulnerable to contagion by the new pandemic of COVID-19 (Alemi et al., 2020). Limited access to healthcare and mental health problems are other issues in play. In addition, there is reason to fear that comorbidity with other infectious diseases such as tuberculosis could negatively impact treatment outcomes. The fear of COVID-19 may also increase discrimination against refugees in the host society. Lastly, most Syrian refugees in Jordan do not have a job to return to after restrictions are lifted.

### Resource Challenges

The global public health agencies’ emphasis on health programs might have been necessary, but may also have led to less attention being put on developing the actual healthcare delivery systems in the targeted developing countries (Razzak & Kellermann, 2002). During a crisis, such as the refugee crisis in Jordan, this deficiency becomes most evident. According to Razzak and Kellerman, the three main functions of any healthcare system are: meeting the expectations of the population, improving health, and protecting society against the many costs of ill-health. They argue that emergency care, including critical care, is a core function of the healthcare system and plays an important role in the financial protection. They name cost as a severe and common obstacle for emergency care in developing countries.

In the first 2 years of the Syrian civil war, the number of Syrian refugees in Jordanian public hospitals had increased by 250% and by 600% for surgical operations (Coutts & Fouad, 2013). Combined with insufficient aid (Balsari et al., 2015; Francis, 2015) and the already fragile Jordanian finances, especially due to the lack of natural resources (Francis, 2015), the situation has inevitably led to resource shortages—leaving the public healthcare system essentially bankrupt according to some (Coutts & Fouad, 2013). This has worsened an already strained socio-political situation, the symptom being a growing frustration among the Jordanian public, who have progressively come to feel that they are paying too high a price (Francis, 2015; Lupieri, 2020). Dumit and Honein-AbouHaidar (2019) has reported that resource shortage negatively affected performance from the Lebanese healthcare system.

### A Postcolonial Approach to Nursing in Emergency Care for Refugees

The postcolonial approach has always been concerned with interrogating the interrelated histories of violence, domination, inequality, and injustice, and with addressing the fact that, and the reasons why, millions of people in this world still live without things that most of those in the West take for granted (Young, 2012, p.9).

The idea of this study took shape based on a long-term political engagement with the Palestinian cause and a conviction that social justice and human rights issues should be an integral part of nursing in general and advanced nursing in particular. According to Mohammed (2006), postcolonialism can be a way of contextualizing health disparities beyond the notion of culture. Our familiarity with the situation of Palestinian refugees and the understanding that many have been Jordanians for generations seemed like an intriguing background against which to explore the nurses’ experiences in caring for refugees, especially in light of the recent Syrian refugee influx. Similar to what Mohammed (2006) expressed regarding her study on American Indians perception of diabetes, the nurses’ narratives and its value were at the center of this project.

Postcolonialism provided a theoretical background for this project as it was well aligned with the stated goals and principles of the study. Mohammed (2006, p.100) writes that postcolonial theory can “reframe knowledge within an analysis of systems of exclusion and the politics of science,” something that had been our goal from the very beginning. Mohammed further states that a postcolonial approach can help (nursing) researchers avoid reproducing injustices and stereotypes, illuminate complexities, and contribute to the construction of a more socially just world. These are high ideals to live up to for a first-time postcolonial effort, but they do describe our intentions well.

Following Halabi (2005) call for more qualitative nursing research on refugees, the delivery of mainly primary care and management of chronic illness for refugees in Jordan have been explored but not extensively so. Advanced care has been explored even less. In the research available, this population and similar refugee groups do appear to encounter barriers to healthcare access regarding education, economics, policy and host community resource limitations. Moreover, the health issues within the populations are numerous and there is evidence pointing to severe gaps in the healthcare response, especially for non-communicable diseases (UNRWA, 2015). Such gaps may cause direct and indirect effects on the preconditions for or even outcome of more advanced healthcare such as emergency and critical care. Nursing and nurses are instrumental in the delivery of this healthcare. Therefore, aiming to contribute to this much needed research area, the study was designed to explore nurses’ perceptions of caring for Palestinian and Syrian refugees in critical and emergency care in Jordan. Consistent with focus of study and methods, the research questions guiding the study were: 1) What are nurses’ experiences as care providers for refugees in the context of emergency and critical care? 2) What practical obstacles are perceived related to resources and organization? 3) What do the nurses appreciate about their work with the refugees?

## Methods

We used a phenomenological lifeworld research approach to enable the study of the lived world of nurses as described by Dahlberg et al. (2009). This approach accords with postcolonialism because it is possible to take power dynamics into account (Mohammed, 2006). Our aim to implement a postcolonial approach beyond a mere theoretical platform was also drawn from a political engagement, most notably with a focus on the enactment of power throughout the project (Kirkham & Anderson, 2002). The study itself represents this active approach to postcolonial inequities by deliberately deferring voice from our privileged positions, including the Swedish nurses we could have interviewed about the refugee crisis.

As Mohammed (2006) instructs in her guide to postcolonial nursing research, every step of the research design was considered a moment of ethical choice. Was the risk of reproducing injustice outweighed by the possible usefulness of insights from such an under-researched field? Could the intentions of the project be transferred through the local facilitator and beyond language barriers? What compromises could be made without losing the integrity of the project? These were some of the questions that we struggled with. According to Mohammed’s instructions (2006), what is also crucial is reflexivity about the researchers influence on the selective observations that form data gathering and the theoretical interpretations made based on those data. For us this began with reflections and discussions about what we thought we knew about the subject, practicing how to formulate the interview questions with as little of that pre-understanding as possible to make room for the nurses’ perceptions rather than our own.

### Participants

The informants were selected through purposeful sampling (Polit & Beck, 2016), based on the connections and knowledge of the local facilitator in Jordan. Inclusion criteria for informants consisted of the following: the nurse should be working in critical or emergency care with Palestinian, Syrian and other refugees in Jordan for at least 1 year and have the ability to speak English with relative ease, as assessed by the facilitator or his connections. Variations in gender and age were considered desirable when the facilitator searched for the nurse informants. The nurses that participated in the interviews were between 26 and 34 years old, seven females and five males, and they had between 3 to 13 years of work experience. Table 1 displays an overview of their demographics. Please note that the nurses working in public hospitals henceforth will be called “public nurses,” and those who had employment in the camps are referred to as “humanitarian nurses” when the distinction is relevant.

**Table 1.** Demographics of Participants.

Workplace	Public hospital A (*n* = 4) including critical care and emergency care Public hospital B (*n* = 4) including critical care and emergency care Syrian refugee camps hospital/clinic (*n*= 3) Field clinic serving the berm and mobile clinic serving urban areas (*n* = 1)*
Nationality**	Jordanian (*n* = 6) Jordanian of Palestinian descent (*n* = 5) Palestinian Jordanian (*n* = 1)
Educational level	Bachelor in nursing (*n* = 5) Master degree in nursing (*n* = 3) Enrolled to master program in nursing (*n* = 4)

* also worked in a Syrian Camp Hospital.

** Self-reported nationality. Only one of the informants presented themselves as “Palestinian Jordanian.” The other five have been given the Jordanian nationality they themselves stated in the first demographic questions, but with the added “of Palestinian descent” as they later in the interview disclosed this. Six of the informants stated Jordanian nationality and did not disclose Palestinian or other descent later on. All of the informants had Jordanian citizenship.

### Data Collection

Data were gathered through semi-structured lifeworld interviews built upon the philosophical assumption of phenomenology, that is, openness and a genuine interest to understand nurses’ experiences of the study phenomenon, described by Brinkmann and Kvale (2014). An interview guide included topics based on the research questions as well as examples of open-ended questions was used. During April 2017, 12 interviews were conducted in English as it was the language best spoken between both the informant and the interviewer. The interviews lasted 18–53 minutes and occurred in the nurses’ workplaces or at the University of Jordan, at the informants’ convenience.

### Data Analysis

The gathered data were subjected to inductive qualitative content analysis, with the aim to achieve a condensed, broad description of the studied phenomenon in unfolding the meanings of the text (Elo & Kyngäs, 2008). Codes with perceived similar meaning were grouped together, and preliminary subthemes gradually emerged through the process of formulating what the codes described. The subthemes then were reworked, collapsed, and renamed as our understanding of them grew. With reflexivity in mind, we backtracked to the original words of the transcripts again and again, trying to stay true to their meaning as well as we could. We did this by asking ourselves what each code meant, how we knew that, and lastly if it could mean something else, all to stay vigilant against quick assumptions. The abstraction process continued as far as was possible given the quality of the data, as well as the time and resources available. Each step of the analysis was discussed and validated among the researchers. To organize the data, open coding was employed (Elo & Kyngäs, 2008). Table 2 presents an example of the analysis process.

**Table 2.** Examples of Analysis Process.

Meaning unit	Codes	Subthemes	Themes
“Yeah, it’s difficult for them to pay these kinds of costs. Yeah. I don’t know how they manage them, so… Really, I’m not going to say I didn’t care, but, you know with a lot of heavy work, you know, a lot of dealing with patients…Your wondering is going be decreased gradually, gradually, gradually…” “Look: I am trying…We are trying... My father says every day for us…(Phone ringing) He says, anything…He says to do anything in the work, stay in the work. Not take it home. Any problem stays at work. Any problem in the home, stays in the home. We are here finished now. Refresh our mind, and go home, never to speak anything about the work. And for my family: what happened at work? Nothing. Good. We want to leave.”	Lack of awareness Hopelessness Stress Keeping the distance Protecting self Keeping the distance Protecting self Separating private life from work Stress Isolation from family	Ethical predicaments Handling the ethical stress Ethical predicaments Handling the ethical stress	Nursing challenges

### Ethical Considerations

First, written ethical approval was obtained from the University of Jordan (1/2017/1409). It was then approved by the public hospitals (MOH rec. 170,057) and lastly, the study received a general approval from the Jordanian Ministry of Health (April 13, 2017/5571), making it possible for us to approach both public hospitals and individual nurses working within the camps, the latter based on the local facilitators’ own connections. The participants gave informed consent after receiving verbal and written information regarding the study’s aim and methods and the voluntary nature of their participation, including the right to withdraw without giving any reason. Confidentiality was preserved by removing identifiers and using code numbers, and the results include no sensitive data that may identify participants.

## Results

Findings are presented as three main themes, namely, understanding of life in refuge, nursing challenges, and nursing opportunities. These each include three to four subthemes; please see Table 3 for an overview.

**Table 3.** An overview of Themes and Subthemes.

Themes	Subthemes
Understanding life as a refugee	• Displacement • Existential crisis • Limited access to healthcare
Nursing Challenges	• Domestic problems • Practical difficulties • Organizational barriers • Ethical predicaments
Nursing Opportunities	• Cultural similarities • Equality in care • Personal growth

### Understanding Life as a Refugee

The nurses empathetically reflected on their experiences of refugees in Jordan and within the Jordanian healthcare system. About half of them reported that they had close relatives who had undergone similar experiences and referred to their familiarity from that perspective. Others spoke of refugee neighbors or other personal connections to the refugee groups in question. The broadness of the theme reflects the openness of the interview questions. The nurses described whatever came to mind about who Syrian and Palestinian refugees are, what they may have experienced before coming to Jordan, and what their life there was like inside and outside of the camps. Perceptions of the refugees’ implications as a patient group, however, varied more among them. Specifically, it varied most between humanitarian nurses and those working in public hospitals. The subthemes below include displacement, existential crises, and healthcare access, as explained by the nurses.

Nurses’ descriptions related to the subtheme *displacement* included a devastating loss of home, loved ones, and assets, leading to a difficult journey involving legal or illegal entry to Jordan. Furthermore, the nurses reflected on why the refugees had left their homes: to escape war, dangers, and assaults, as well as to protect themselves and their families’ lives. The nurses expressed sadness concerning the difficulties in neighboring countries and sympathy with the refugees. Although safer than war, life as a displaced person in Jordan was described as an ordeal. Poverty, poor living conditions, and a lack of livelihood, education, daily necessities, and other desired life opportunities were mentioned by the nurses when asked about the living conditions of the refugees for whom they cared. In this regard, the nurses also made a clear distinction between Syrian and Palestinian refugees: Syrians were considered to be refugees, whereas Palestinians were no longer considered refugees. *“*Maybe because they [Syrian refugees] came to another country, they had no money, no essential things, and no education. Like the Palestinians in the past.” (Interview 12)

The nurses further described life for refugees in Jordan within the subtheme of *existential crisis*. The nurses perceived the refugees as vulnerable in many ways, since they had lost so much of their lives to conflict. Consequently, they could sometimes be perceived as socially isolated and frustrated about what their lives had become. The refugee patients living in the Syrian refugee camps also would have to deal with the loss of freedom of movement. Lastly, the nurses recognized that the refugees were exposed to prejudice, fear, and anger from the host community. The nurses described how the refugees brought this feeling of not being welcome into the hospital or clinic where they sought care, and how that could influence their first encounters with the healthcare staff. The nurses had also noted some structural inequalities for refugees, related to socio-economic status, legal rights, and opportunities. Here too, the nurses stressed the differences between the refugee groups. Initially, they would say that Palestinians enjoyed their full rights in society and that Syrian refugees mostly lacked them. However, when this was explored further, it led to the discovery of issues such as a lack of citizenship among some of the Palestinians, as well as more complicated policies specific to Jordanians of Palestinian descent.

Regarding the subtheme *healthcare access,* the nurses expressed awareness that the lack of money and health insurance combined with expensive treatments could be challenging for the refugees. The nurses also described the refugees’ process of care-seeking as complex and difficult. In some instances, it was so complex that the nurses themselves were unable to completely narrate it for the interviewer. The nurses who could do so explained that in the closed camps, the level of healthcare was limited to the primary, and that it took time to get external referrals for more advanced care or to handle long-term health issues. However, at least basic levels of healthcare were considered relatively easy to access within the camps. Many more refugees live outside camps, which most of the nurses knew, and they acknowledged that non-camp refugees could have difficulties accessing even basic healthcare. Public hospitals were described as overcrowded and understaffed, which negatively impacted access there; they explained that only a few mobile clinics were around to serve those unable to access the hospital at all.

Maybe the Syrian refugees in the camps have advantages in healthcare. Because at this camp, all the Syrian refugees, maybe 40,000 refugees in [name] camp, are there. We have a big hospital at [name]. The team there, all people should have care. … And there are sponsors for Syrian refugees, and they ensure good communication with them. Outside, in Jordan overall, in the public hospitals … okay? The process takes a longer time. You must come and visit the doctor and [go through] … many [administrative] processes. (Interview 8)

### Nursing Challenges

The nurses shared stories revealing that they were severely affected by the refugee crisis, both privately and in their profession. They are nurses, and they wanted to provide care. The subthemes domestic problems, practical difficulties, organizational barriers, and ethical predicaments illustrate why and how they were prevented from doing so, how that made them feel, and how they tried to handle these feelings.

The subtheme *domestic problems* refer to how the conditions in Jordan were directly connected to challenges in delivering care, according to the nurses. The data revealed an intense awareness of their country’s limited natural and economic resources, as this was highlighted by all nurses.

Increase the nursing, or all medical teams … all of the medical team. And we need … more equipment to work with patients. The ER building, it’s a building from the past, for about 200 or 100 patients… But now we have a lot of patients. We need …. a large building. We need more medical teams. […] About this—this is for us and the country, not for just us and the work we are doing. If there is something to do, we are doing it. No problem. But to decrease the load for us … to be [able to go] back home, happy. (Interview 6)

The nurses’ other explanation for Jordan’s difficulties was how the great number of refugees exerted an unbearable burden on their already struggling country and communities. They explained how the competition for work had created economic instability, with rising costs and shrinking salaries, including for themselves.

Because we don’t have a lot of vacancies, you know. But for Syrians who work illegally here, they get less money, so the manager or the shop owner or the restaurant owner, they start to take … two Syrian people rather than one Jordanian. (Interview 4)

Other issues raised under the subtheme *practical difficulties*, in addition to equipment shortages, came directly from the refugee burden which was creating an enormous load in public hospitals. Consequently, intensive care unit (ICU) nurses had many critical patients and very limited time with each patient, which contributed to the difficult working conditions. Working in the Berm, an area between the Syrian and Jordanian border harboring Syrian refugees not allowed into Jordan (Amnesty International, 2016), as a humanitarian employee was described as dangerous and entailing barely any equipment.

The nurses identified a lack of health knowledge as another practical barrier, making it difficult to perform both treatment and health education. Other psychosocial challenges mentioned included anomalistic gender cultures and aggressive behaviors among patients or their families.

They look at health as if …. If it’s pain, I do not have to go see the physician. I don’t have to do any check-ups. For the pregnant women, you see sometimes the woman just delivered, and she didn’t know what was inside her tummy. (Interview 4)

The nurses identified several more issues related to the subtheme *organizational barriers*. In the larger context, the nurses working in the humanitarian field perceived a lack of coordination among the nongovernmental organizations active in Jordan, while the nurses working in public hospitals pointed to the differences between the public and private healthcare sectors. These differences affected the nurses’ working conditions and created inequalities among patients, particularly in their ability to access more expensive private care, or care period, as fees were always charged for healthcare outside the camps. A lack of nursing research to advance nursing science, as well as communication issues and obstructive gender culture, were other structural barriers that they mentioned. In the humanitarian field, the organizational barrier for nurses was gaining less experience, as the refugee-specific health aid was merely basic. The nurses also had less secure employment since NGOs had short-term contracts with the Jordanian government and therefore could only make hires for shorter periods of time.

Firstly, we have primary… And sometimes only secondary equipment. We don’t go to the tertiary. Something like cardiac arrest, … we should address it as primary care healthcare. Start compression, and make Ambu Bag, and wait for them, wait for the help to come. This is our work. But in the desert … you don’t have electricity. (Interview 9)

The nurses working in the public hospitals had to deal with other organizational issues, such as a more traditional staff hierarchy and a lack of possibilities to change their work situation as nurses. They also expressed that the lack of time off diminished their opportunities to attend training sessions or even to think about such matters.

When you come as a newly graduated [nurse], … you have new knowledge, new information, new ideas. But then, he [the doctor] abruptly stops you. Don’t change. Don’t do anything. We don’t want to change. That’s what he told you. If you want to change, and say, “We must change. It’s important,” you are a troublemaker. (Interview 10)

In summary, the nurses called for changes in the organization of healthcare for refugees in Jordan. On a larger scale, they highlighted a need for coordination between NGOs in the humanitarian field and suggested the United Nations. They wanted to see health education delivered to smaller groups to achieve a greater impact, and they suggested support groups for refugees. They argued that the most vulnerable refugees needed better health access. The nurses emphasized the need for attention concerning infections, especially in the closed camps. Preventing complications from chronic diseases was also a challenge they wanted to address. A connection between the need to improve triaging and prevent outbreaks of communicable diseases and complications from chronic diseases was also made. Lastly, the need to secure the arrival area in the Berm to allow for the safe delivery of healthcare there was also highlighted.

In the public sector, there was a demand for more empowerment, appreciation, and authority. They reasoned that they could acquire these through more extensive leadership courses in their training program, a longer program introduction, and more training in general. They also pointed to a need for changes in nurses’ attitudes and practices and for a more family-centered nursing practice in Jordan.

New instruction in nursing. New research. More research. More ideas about nursing as an art or science. You can do more than what you can do now. You can be a decisionmaker, not just a machine. He [the patient] comes to ER. The nurse knows what he has; the doctor doesn’t know, you know? The nurse asks the doctor to do what he must do, you know? You must appreciate the nursing art more than what we… at the same time. I hope. (Interview 10)

*Ethical predicaments* were identified in the data as a subtheme involving feelings of stress, desperation, hopelessness, and frustration. The nurses seemed to simply feel overwhelmed with responsibility. As healthcare providers for refugees, especially in the humanitarian sector, they expressed feeling exceptionally alone in the world. They also exerted immense pressure on themselves to not make any mistakes and to show the refugees that at least someone cared, but they still seemed to feel inadequate. They described feeling torn between the refugees and the Jordanian public. Their friends and families did not always understand or support their work choices. The nurses believed there was insufficient time for a truly holistic nursing approach. Furthermore, they expressed mixed feelings about the refugees, as they themselves made personal and financial sacrifices to keep their country afloat while overloaded with so many people in need. The public-sector nurses sometimes felt trapped and forced into their work situation, as they had not intended to work with refugees under those circumstances.

When I was an undergraduate student, I was a solid person. Solid. I was just doing my duties. I had very proud friendship relations. But today, I have few friendships […] because the perspectives of my friends regarding Syrian refugees differ from my own perspective. We discuss all of these issues. … I try to not discuss Syrian refugee issues with my friends. But sometimes we discuss them because I’m working, and I come telling them about them, … so we discuss them. Many of them are seeing things differently than me. (Interview 3)

To handle the strain of their situation, the nurses employed several strategies. They deliberately distanced themselves from the refugees’ situation by attempting not to engage personally, or they even had a clear goal to separate their private life from work. They described receiving this advice from both family and employers. They distanced themselves partly through an intentional lack of awareness, striving not to think excessively about or problematize the situation. Statements comparing other countries as worse or more foreign than the refugees were also utilized, seemingly to relieve the nurses’ conscience. Notably, the nurses of Palestinian descent also seemed to distance themselves from the refugees, focusing on the suffering of their country. “I will tell you a thing: … I am Palestinian. Ah, but I have a national number. We are very, very happy in Jordan. It is a good country. It needs help to … not to become like other countries.” (Interview 12)

### Nursing Opportunities

Critical care and emergency care were described as different from other healthcare departments. Moreover, the nurses believed that they and the refugee groups in question had cultural advantages that refugees in other parts of the world lacked in relation to their care givers. The public and humanitarian fields had different opportunities available to them. These perceptions are delineated in the following three subthemes: cultural similarities, equality in care, and personal growth.

The subtheme *cultural similarities* captured nurses’ views on Palestinians living alongside Jordanians for a prolonged period, along with shared geography and a common history. Syrian refugees did not share history with the Jordanian population in the same way, but they were considered to be culturally similar to both the Jordanians and Palestinian Jordanians, which in some ways made it easier to share a country and give care to them.

The subtheme *equality in care* is based on the nurses’ perception that equality is better within emergency and critical care than the rest of the healthcare sector. They were often aware of and able to describe the difficulties with which the refugees struggled in the healthcare system. However, regarding their own work in the emergency room and critical care unit, they emphasized the notion that the refugees were treated no differently than others. If a life needed saving, all efforts were made. The physical care was the focus, simplifying all such situations. “You don’t know his nationality. Do you know? Every case that comes here, we receive it. OK? No, there is no…We can’t know if you are from Palestine, you are from Syria…We don’t know.” (Interview 10)

*Personal growth* as a subtheme was exemplified by financial gains in the humanitarian sector, as NGOs paid better than the financially strained public sector. The nurses in the humanitarian field further received refugee-specific training and leadership experience and felt that they could exercise more independence as nurses compared to a public hospital employment. They also thought it was useful to see and learn about health issues beyond those that were common in the Jordanian population. They appreciated getting to know international colleagues, how they worked and their culture. The nurses pointed out that this cooperation also improved their English skills. They perceived it as easier to get involved and care about refugee patients than during their experiences in private and public hospitals. They thought the humanitarian work felt more meaningful and provided better opportunities to do more for the patients:

Sometimes, I was the team leader for 10 doctors and maybe 15 nurses. This gives you skill and experience with how to work with people, how to lead people. And this is the way it works for everyone in the [humanitarian] field. (Interview 8)

Through caring for refugees, the humanitarian nurses expressed that they had learned to care more about people in general. They described personal growth from getting to know the Syrian volunteers and feeling closer and more sympathetic to the Syrian people through them. The nurses’ work experiences had changed their perspectives on life in many ways and developed their sense of responsibility, solidarity, generosity, and kindness. They assessed that they had improved their communication skills and learned to handle stressful situations better. Drawing on their insights from meeting the refugees at work, they tried to coexist with the refugees in their society, even though they sometimes found it difficult.

Before I worked with them, I was a little bit fragile, but after I worked with them, I saw how they are strong, as women and children, and they claim their rights, even in the camps, after all these problems that have happened to them, so I became very strong. And I like…I became strong in the camp, but after two years, I have a lot of burnout because it’s too stressful to talk about problems and every day, daily life. So, you go to work and hear about problems and then go back home…Some problems come without you noticing, so it’s overwhelming. (Interview 4)

The nurses in the government hospitals on the other hand, highlighted their self-confidence from handling difficult medical situations. In the ICU, they felt they had learned to get more involved in the patients’ overall situation. However, they did not consider the refugees to be a specific group from which to draw experiences, so they could not express any such experiences. Lastly, the humanitarian and public-sector nurses did describe one shared opportunity: for them to represent Jordanian society to the refugees, or a version of the society that provides care:

Yes, the responsibility is higher than in the past. The caring process, the kindness, the communication process with these people. You should be more than good in this type of situation because you’re the only one reflecting the humanity, a picture of Jordan and the world for these people. You should do as much as you can. (Interview 8)

## Discussion

The nurses’ perception of what it means for their refugee patients to be displaced in general and specifically in the Jordanian society and health system, could be described as a struggle. Refugees were perceived to be poor and healthcare expensive. The nurses also stated that difficulties in healthcare access differed between camp residents and non-camp refugees. They highlighted communicable diseases, chronic diseases, health education and coordination between different care actors and healthcare levels as areas of concern. They expressed acute awareness of the lack of resources in their workplaces as well as in their country and experienced that the Jordanian public had become fed up with the situation.

There are three main findings from this study that contribute new insights. The first finding relates to nurses’ perceptions of the inherently equalizing nature of emergency care. The ER and ICU nurses were united in their perception that if the patient comes there, they get what they need. Paradoxically, the second main finding highlighted the important differences between the experiences of the humanitarian and the public nurses. Public nurses had not chosen to work with refugees. And they did not express a general awareness about the refugees as having special needs, even though the public hospitals are where all advanced care takes place. This would seem like a barrier to proper care, particularly in the ICU where stays are longer. The third finding offering new perspective was related to the nurses’ ability to assess their patients living conditions; no Palestinian Jordanian was considered to be a refugee, even those that lived in refugee camps and lacked citizenship.

Open commitment to critiquing of the status quo and building a more just society is considered one important feature of the postcolonial research method (Kirkham & Anderson, 2002). Those are the shared goals of this study, and thus critique of the economic world order seem important to include. Addressing capitalism has been pointed out as one of the weaknesses of postcolonialism or rather its scholars, in the tendency to take capitalism for granted and by doing so, seeing it as a neutral background (Mezzadra, 2011). However, to achieve social justice and the elimination of health disparities, radical change regarding political and economic systems will be necessary according to (Mohammed, 2006).

Economy and resources seem to present one of the clearest connections between colonial history and postcolonial reality. To illustrate this line of thought: consider that globally, significantly more resources are put into the conflicts causing the high refugee flows than aid for those in need (Stockholm International Peace Research Institute, 2004, 2016). As previously stated, most refugees end up not with their former colonizers but in other poor countries or ex-colonies. In 2016, Jordan spent $870 million annually on Syrian refugees alone. This is equivalent to 5622% of GNP in traditional donor terms (Oxfam International, 2017). Meanwhile, the UN aim for aid volume is 0.7% of GNP. Sweden contributes with around 1% of its GNP to foreign aid and that is a relatively high number compared to other rich nations (Government Offices Of Sweden, 2015). While host states (generally poor and neighboring ones) are hindered from sending refugees back where they may face persecution, other countries (specifically the rich ones far away) are not in any way obliged to share the burden financially or by resettlement (Lupieri, 2020). This would seem to uphold the status quo in power dynamics rather than offering solutions for change and self-realization, a typical trait of the white supremacy (Willer, 2019) that is closely connected to colonialism.

The nurses verified the disquieting notion that the Jordanian public had developed a fear of losing what they have and frustration about what they already sacrificed, resulting in anger towards the refugees, as previously described by (Francis, 2015). Lupieri (2020) similarly wrote about when “brothers and sisters become foreigners,” but added another take on the role of the Jordanian government. Lupieri suggested that health policies for refugees had become a battleground between donors, multilateral institutions, and the host states. She further argued that this can be seen in how the reduction of health services for Syrian (and other) refugees in Jordan sometimes has coincided with more international donor financing and other times with less. The conclusion being that influencing factors other than financial assets have been at play during these last 10 years, such as an unwillingness to permanently integrate the Syrian population with the Jordanian society, concerns about the nations’ stability, and efforts to calm a resentful Jordanian population.

The hardship of low- and middle-income host communities has been noted by the European Commission (n.d.), and the nurses connected challenges in their daily work to that larger picture. The difficult working conditions they all faced would bring incredible moral distress, as described by Oh and Gastmans (2015). The nurses did express feeling stressed if they were aware that the refugee patients had special vulnerabilities and complex needs. This moral distress sometimes generated frustration due to their limited ability to meet those needs. Findings concerning frustration and stress have been related to work in humanitarian assistance previously, for example in research involving Swedish healthcare professionals (Bjerneld et al., 2004). Interviews with Lebanese nurses by Dumit and Honein-AbouHaidar (2019) explored these issues more deeply exposing varying degrees of fatigue, burn out, and depleted compassion leading to a rationed nursing practice.

Another finding related to refugee research conducted elsewhere was the nurses’ thoughts about the (Syrian) refugees’ lack of health education—or cultural differences in relation to healthcare-seeking behavior, depending on the viewpoint. A Swedish study on the same issue emphasized the urgency to develop models of refugee care based on appropriate organization structures and national policies to meet the need for health education (Hultsjö & Hjelm, 2005). These clinical implications most likely are relevant in Jordan as well.

An incidental but seemingly important finding was how the nurses did not consider Palestinians to be refugees. Not mentioning citizenship status, they generally assessed Palestinians to have equal social and legal opportunities to Jordanians. This view differs from the picture painted by the literature. To summarize: UNRWA has administrative responsibility for more two million refugees in Jordan and still serve refugee camps for some 370,000 Palestinians in substandard living conditions. Those of Palestinian descent have very little governmental representation (Francis, 2015), and one in four do not have the opportunity to obtain Jordanian citizenship (United Nations Childrens’ Fund, 2016). However, the interviewed nurses of both Palestinian and non-Palestinian background repeatedly stated equality, shared opportunities, and general unity among Palestinians/Palestinian Jordanians and Jordanians. Why did they? Perhaps these circumstances identified as problems in research and by NGOs were not present in the minds or everyday life of the people. Admittedly, this is also likely a more complicated political issue than could be properly dealt with in a single interview, particularly when meeting the informants for the first time and as a foreigner speaking their second language. Tendency to avoid political subjects has been studied by Achilli (2014), who found that Palestinians living in a Jordanian refugee camps tried limiting their involvement in anything political, as it is a “sphere where friends and enemies are distinguished.” In regard to the Palestinian–Jordanian identity, a nonpolitical everyday life helped them handle the complexity of their situation. To allow for these two differing accounts of who can be a refugee, recently arrived Syrians but not Palestinians even if they do live in a refugee camp is something that Mohammed (2006) highlights as important for the postcolonial approach. Presenting outlying accounts in general could help further the important problematizing of the phenomenon according to her recommendations. She further writes that the role of history and structural inequalities in health disparities often is overlooked when simplistic views on culture dominate, which might eradicate history and even continue colonial injustices. This would seem to be applicable to the situation of Palestinian Jordanians, from the perspective of this study.

It could seem counterintuitive to address Palestinian refugees with postcolonialism on account of the ongoing occupation, and the subject of Palestine has been rather absent from postcolonial discussions. However, there are also more recent voices arguing for its relevance (Williams & Ball, 2014). They suggest that however complicated and politically difficult, Palestinian cultural expression does belong within the postcolonial field, as it has come to be about much more than the postcoloniality which Palestinians have not yet reached (to the extent that it has been reached elsewhere). As a framework for analyzing expressions of inequality, oppression and struggle, Palestine and Palestinian refugees will hopefully be given much more postcolonial scholarly attention in the future. The scope of this study was only partly on Palestinian refugees, and our own capacity for nuance is clearly limited, but we would argue that however thinly represented, the choice of a postcolonial approach is even more motivated by our focus on this specific refugee group.

The most evident specialty-related finding is the data in the subcategory of equality in critical care, including emergency care. The public nurses made it very clear that patients were met with little distinction on this level of care, rather it was simply about saving lives and fixing physical problems. The nurses explained that this could also be seen in healthcare policy; refugees faced many difficulties accessing healthcare, but less in the case of acute illness according to them. There may be an equality enhancing trait in emergency care, as the nurses claimed, but it could also represent a hidden risk for an even greater lack of holistic awareness than the obstacles already identified by the nurses. According to previous research, access to emergency care and critical care for refugees does have certain barriers; knowledge of how and where to seek care is generally lacking in the Syrian refugee population camps, and there is an administrative process required for admittance (Dator et al., 2018). Above all, cost is a barrier for all refugees coming to public hospitals (Al-Rousan et al., 2018; Dator et al., 2018; Doocy et al., 2015). This could lead the nurses to incorrectly assess the accessibility of their field; everyone admitted perhaps receive help, but does everyone who needs it find their way there? Another possible explanation for the apparent lack of holistic critical care nursing is the absence of requirements for specialty training for nurses in critical care in Jordan. In fact, all the interviewed nurses in the ICU of the public hospitals lacked specialist degrees. It is therefore difficult without further research to draw further conclusions about the main origin of this finding.

When asked about what they gained from or liked about working with refugees, the public nurses only answered in general terms about their area of nursing. While they could relate to the refugees on a societal level, they generally had no view on the implications of refugee patients regarding their nursing practice. They certainly had experience treating refugees; the public hospitals were filled with them, but they seemed to have reflected little on whether refugees might have specific healthcare needs or what different experience they could gain from the refugees compared to other patients. The humanitarian nurses, however, saw specific opportunities in their field. Higher salary was not the only gain; they also described personal- and skill-related development. They even seemed to cherish the new viewpoint to life that the refugees had given them. Much like for the Swedish health professionals (Bjerneld et al., 2004), the frustration and stress from caring for refugee patients was paired with a sense of satisfaction and meaningfulness.

Dumit and Honein-AbouHaidar (2019), sharing findings of exhaustion among Lebanese nurses and negative effects on the healthcare provided by them, stress the need for adequate response to such situations as the ones in Lebanon and Jordan. They conclude that access to care must be ensured for the refugees, paired with increased human health resources. Furthermore, they emphasize the need for proper training in the handling of refugee-specific health conditions. They, like us, argue the importance of reporting challenges and resilience of health workers such as nurses facing a refugee crisis. Lastly, they suggest the need to recruit nurses for policy making, which was suggested by our nurses as well.

### Methodological Reflections

It is of course problematic when a white, European researcher conduct English interviews in an Arabic speaking country while claiming to adhere to a postcolonial approach. The rationale beforehand was that no studies on this seemingly important subject could be found and therefore a white savior (Willer, 2019) type of researcher would be better than no research at all. As the process of unlearning racism has continued, it is now clear that the impact of this study could be considered a performance in white goodness or charity, rather than the act of power transfer it was intended to be. We considered not to move forward with an article transcript, concerned it might be more harmful than helpful, but decided it would have been worse to be given these nurses time and knowledge and not even try to convey their message.

The interview situation is always based on power asymmetry (Brinkmann & Kvale, 2014), but in this instance it is likely to have been even more of an issue due to cultural differences and inequality between the respective countries of origin. As a European in Jordan, the researcher clearly received special treatment. The very existence of this study depended on that inequality, particularly regarding the swiftness of the approval process, and it is likely that these larger circumstances in some ways affected the interview situations as well. The researchers’ efforts to counteract do not ensure that the unwanted power dynamic was averted.

A limitation to the selection process was regarding the inclusion criteria about good knowledge of the English language, which seemed less possible to give significant attention to when the local facilitator arranged the interviews. On the other hand, assuming that speaking advanced English could be associated with a higher social status or level of education, always choosing primarily on the basis of language skills could have excluded voices that now have been heard. Nevertheless, the lack of a common, well-commanded language sometimes made the interviews very difficult to conduct in depth, which inevitably affected the findings. In hindsight, more effort could have been put into finding a solution using translators, since this could have been equalizing in regard to power dynamics as well by not taking their ability to express themselves in their native language away from them. However, the speed with which choices had to be made in order for the interviews to occur did not leave much room for this possibility. Furthermore, the somewhat sensitive subjects of the interview made it seem difficult to make sure that the informants would have felt safe enough to express their opinions if someone local was translating.

A phenomenon that occurred during the interviews that raises the question of power asymmetry is the way the informants (pre-interview) answered the question about their nationality. As noted in the method section, half of the informants had Palestinian roots. Only one of them mentioned this before the interview started, by identifying as “Palestinian Jordanian.” The others simply said they were Jordanian. Later in the interview; however, they would talk about their Palestinian descent. Perhaps there is no meaningful reason behind the informants’ different courses of action. They could have simply answered by stating the nationality they identified with, and when another question made them feel as though that was relevant, they disclosed their Palestinian roots. But perhaps this was an effort for gaining counter-control (Brinkmann & Kvale, 2014), in response to the inequality between them and the researcher? Or could this stand for an alienation of Palestinians in the Jordanian society, illustrating the complex political issue of the historical and current refugee-situation in Jordan? Nonetheless, it is a limitation that the preparations of the study did not include a clear approach to the fact that some of the nurses interviewed would in fact be “Palestinian Refugees,” at least by the UN administrative definition.

In summary, these reflections are about being an outsider in a field of research that is by nature influenced by whichever connection the researcher has to the context and the participants, both during data collection and analysis (Brinkmann & Kvale, 2014; Dwyer & Buckle, 2009; Elo & Kyngäs, 2008). There are arguments for the notion that there are in fact equal advantages and challenges to being an outsider as a qualitative researcher (Dwyer & Buckle, 2009). It could perhaps be argued that the researcher to some extent touched the more ambiguous space in between being an outsider and insider, as a fellow nurse.

### Future Research

Our suggestions for future research on emergency and critical healthcare for refugees in Jordan include conducting interviews with humanitarian and public nurses separately, or together with a more structured comparison to chart their different realities and perspectives. The apparent lack of a holistic approach in critical care is disconcerting considering the complexities that the refugees bring with them into critical care units and emergency rooms in Jordan. It suggests a need for further research on the subject of critical care for refugees in other countries as well. Above all, research in this area in particular should be conducted by scholars native to the culture and language, as they would be far more qualified to reach a deeper understanding of the situation.

## Conclusion

Even in this somewhat postcolonial world, the inequalities of colonization are still present. The fact that Jordan still stands as a nation at all is a testament to incredible resilience; let it be unsaid here whether this is attributable to the state or to the people. In the light of their struggle, the cries of “refugee overflow” from European countries such as Sweden during the most intense period of displacement would be almost laughable if they were not so privileged. Morally, we all have the responsibility to help those in need, whether in the ICU, our neighborhood, or another part of the world. Organizing and executing healthcare for the most vulnerable is also a fundamental part of the nursing field. The stories shared in the perspectives of the nurses in this study raise questions concerning what long-term effects of intense human suffering in this region may cause on an interpersonal level as well as globally.

Why should refugees live in detention in camps anywhere, or in poverty in urban ghettos? Why should Jordan and a few other countries bear the brunt of the refugee crisis to the extent that nurses can no longer afford their own living expenses? Why should these nurses have to work with too many patients, too few colleagues, and too little equipment? They should not. We should all share the burden. The answer to why is solidarity. As an intended act of solidarity as well as an exploration of an under-researched area, this study has strived to convey the voices of nurses doing what they can in a difficult situation. Let us listen to them, and let them lead the way.
